# Assessing relative post-release mortality for the transparent goby fishery: Environmental drivers and the utility of vitality metrics

**DOI:** 10.1371/journal.pone.0230357

**Published:** 2020-04-09

**Authors:** Maria del Mar Gil, Miquel Palmer, Gabriel Morey, Amalia Manjabacas, Elena Pastor, Carlos Díaz-Gil, Antoni Maria Grau

**Affiliations:** 1 Laboratori d’Investigacions Marines i Aqüicultura (Balearic Government), LIMIA, Port d’Andratx, Balearic Islands, Spain; 2 Department of Ecology and Marine Resources, IMEDEA, Institut Mediterrani d’Estudis Avançats (CSIC-UIB), Ichthyology Group, Esporles, Balearic Islands, Spain; 3 DGPMM, Direcció General de Pesca i Medi Marí (Balearic Government), Palma, Balearic Islands, Spain; 4 ICM, Institut de Ciències del Mar (CSIC), Barcelona, Catalonia, Spain; Department of Agriculture, Water and the Environment, AUSTRALIA

## Abstract

The target species (*Aphia minuta* and *Pseudaphya ferreri*) of the transparent goby fishery on Mallorca Island (Balearic Islands, western Mediterranean) are currently discarded when the maximum daily catch is exceeded or when the sorting process is unworkable. The mortality suffered by this discarded fraction remains unknown, although it may be important for resource management. Accordingly, the aims of this study were to (1) assess the environmental drivers of the relative post-release survival of the discarded target species and (2) test the correlations between post-release survival and two behavior-related variables (swimming speed and its increase after a stimulus, assessed via video recording). To do so, mortality of the target species from 47 hauls sampled under normal fishing boat operations was monitored when the gear was onboard and after a few hours. At the reference level (an average depth of 25.7 m and temperature of 15.7 ºC), the immediate survival was 99.9% (95% CI: 97.9 to 100%), but the estimated post-release survival decreased to 47.2% (33.8 to 65.8%). Relative post-release mortality doubled when the water temperature increased by 2.8 ºC or when the fishing depth increased up to 32 m. Furthermore, the swimming speed of the target species was significantly correlated with the estimated post-release survival; thus, this vitality metric may offer a promising strategy for more easily estimating post-release mortality in other fisheries.

## Introduction

A large proportion of global commercial fishery catches are discarded and returned to the sea due to regulatory, economic or other reasons [1]. Discards constitute a waste of natural resources, and they negatively affect both the sustainability of marine ecosystems and the economic viability of fisheries [2, 3]. Therefore, the problem of discarding in European fisheries has received increasing public attention [4], and the current Common Fisheries Policy promotes measures for the reduction and/or elimination of discards (Regulation EU No 1380/2013 of the European Parliament and the Council on the Common Fisheries Policy).

The implementation of ecosystem-based fishery management and the need to restrict harvest levels have highlighted issues relating to discard mortality [5, 6]. Therefore, to fully assess the effects of fishing at the individual, population and ecosystem scales, it is necessary to quantify the levels of discarding and associated rates of fishery-induced mortality [7]. The fraction of the catch that may survive when released is highly variable, depending on species, gear, depth, water temperature or fish size [5, 6, 8]. In some cases, very high survival rates have been reported [9, 10].

However, properly estimating discard survival is challenging. Estimating survival from the proportion of catches that are alive when onboard (i.e., assuming that any released fish will survive) has been demonstrated to be a naïve approach [11]. A larger post-release mortality rate than expected according to the survival observed at the time of release could occur because released fish can suffer sublethal effects (e.g., masked injuries or barotrauma) that may eventually result in delayed mortality from predation, physiological stress, or disease [12]. In fact, post-release survival (i.e., the long-term survival of the fishes subjected to the fishing process and subsequently released to the sea) is reduced by both immediate mortality (i.e., the mortality when the gear comes onboard) and delayed mortality (i.e., the mortality suffered after fish have been released as a result of the fishing process). Accordingly, one of the most commonly used approaches for assessing post-release mortality is to monitor mortality by keeping a sample of the catch in captivity, although this method can introduce some potential biases and has some logistical difficulties [13]. Tagging- or telemetry-based methods can be alternatively implemented to estimate survival, but they also have drawbacks, mainly related to tag size and tag-induced effects [13, 14]. Alternatively, the use of several measures of activity, reflex response or injury level as proxies of post-release survival has also been proposed [13], and this method has proven useful in some cases, as well as producing rapid results and being low cost [15].

The mortality observed in captivity can be attributed to fishing-related mortality and handling-related mortality. Therefore, in the absence of control fish (i.e., individuals that were not subject to the fishing process but have experienced the same handling and captivity protocol), the cause of mortality cannot be determined; however, genuine controls are unfeasible in many, if not all, cases [14]. Therefore, mortality relative to some reference level is often assessed instead [14]. This possibility is certainly relevant because it allows an accurate evaluation of the putative drivers of post-release mortality. The factors that may affect discard mortality are diverse. For example, gear type or other technicalities related to the fishing strategy, such as catch size, on-deck handling practices or duration of air exposure, are likely relevant [9, 16–18]. The inability to avoid predators may also severely reduce post-release survival [19]. Finally, several environmental factors, such as temperature, sea conditions, depth-related barotrauma and biological factors including species, size, physical injuries and physiological stress, have been reported to affect post-release mortality [6, 9, 16]. Proper assessment of the role of all of these drivers may allow enhancement of post-release survival by modifying fishing practices.

According to all of the above, and as a proof of concept for a strategy that could be applicable across diverse fisheries, the aims of this paper are twofold. First, the effects of two environmental variables (depth and temperature) on post-release survival were estimated via asymptotic extrapolation of data for short-term-monitored fish in captivity. Although the use of survival modeling is well established [16], here, we propose a time-varying mortality model and apply a Bayesian fitting approach that allows us to explore the effect of a number of covariates on the survival rate. Second, given that monitoring fish mortality is difficult, we demonstrated how to extract new vitality metrics based on video-recorded fish mobility. Finally, we evaluated the correlations between these new metrics and the estimated post-release survival to demonstrate the utility of the metrics. This methodology was applied to the target species (*Aphia minuta* and *Pseudaphya ferreri*) of the transparent goby fishery in the Balearic Islands (western Mediterranean).

## Materials and methods

The transparent goby fishery in Mallorca is a small-scale fishery that uses special purse-seine nets over sand and gravel bottoms [20]. The surrounding net is hauled at depths reaching 30 m inside bays during winter. The fishery is economically important, with an average annual gross revenue of 274,498 € on Mallorca Island [21] and high market prices (from 20 to 40 € per kg). The main target species is *A*. *minuta* (a 1-year-lifespan pelagic goby less than 60 mm long) [22], but another similarly sized Gobiidae (*P*. *ferreri*) is also caught. Due to their small size and great resemblance, these two species are usually marketed mixed under the commercial category of the most abundant species. These target species aggregate in shoals close to the bottom (5 to 40 m depth) of bays during the fishing season [23]. Similar fisheries are operated along the Spanish and Italian coasts.

The fishery operates with specific licenses, gear controls, and closed seasons. A management plan was established in 2013 in accordance with European Union rules (Decree 44/2013). The total maximum catch per year for this fishery is 40,000 kg, usually 30 kg/day/boat for *A*. *minuta* and 50 kg/day/boat for *P*. *ferreri*. Landings can occur in only 11 fixed ports, and only 35 boats can operate with specific nets from December 15^th^ to April 30^th^. In addition, a co-management committee was created with the participation of the public administration, fishers’ associations, researchers, and nongovernmental organizations. This committee periodically revises the daily catches and the fishing effort and, if required (for example, if the monthly threshold is not reached), implements more restrictive measures (i.e., closing days) for sustaining the sale price and resources [24].

The discarded fraction accounted for in this fishery is small (not exceeding 10% of the total catch) because sorting fish is technically difficult. Therefore, when discards are over a certain threshold, the entire catch is released (i.e., slipping); thus, such released catches remain unaccounted for in the maximum daily catch. The discarded fishes, i.e., those returned to the sea when the maximum daily catch is exceeded or when slipping is carried out, have an unknown survival rate.

### Sampling

**Fig 1:**
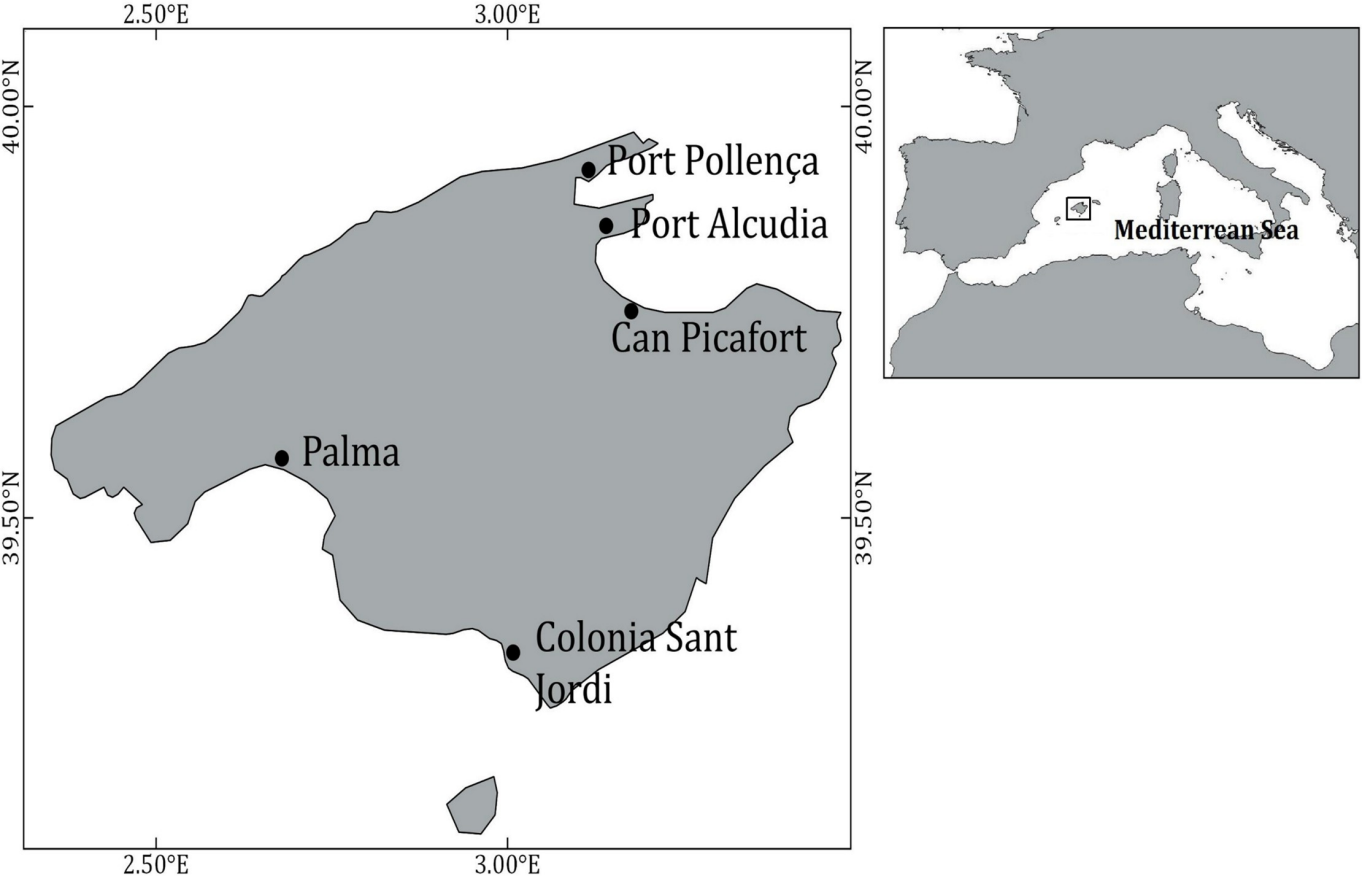
Surveyed area and main ports of the transparent goby fishery of Mallorca Island (Western Mediterranean).

A total of 19 sampling days were randomly selected during the fishing season from December 15^th^ 2015, to April 30^th^ 2016. Between one and three boats were selected at each of the five ports harboring most of the transparent goby fleet of Mallorca Island (Fig 1). A total of 47 hauls were sampled during this study, and usually 2 or 3 hauls were sampled on a given fishing trip. The trips were conducted under normal fishing operations: the mean duration of the tows was 35.1 min (range: 21–55 min), the mean towing speed was 0.5 knots, the codend mesh size was 3 mm, the mean fishing depth was 25.7 m, and the mean water temperature was 15.7 ºC.

For each sampled haul, both short-term survival monitoring and video recording (outlined below) were conducted. Fish (a mixture of *A*. *minuta* and *P*. *ferreri*) were collected just as the codend was brought onboard (*t*_*0*_) by an onboard observer. A random sample of fish was placed into a 15 liter plastic container with sea water and continuous aeration. The time of day, date, location, tow duration, sea conditions, depth and water temperature were recorded. Dead (unresponsive, immobile, and nonreflexive) animals were counted and separated from those that were alive in the container at *t*_*0*_.

At the end of the fishing trip, the containers with the collected samples were landed at the port. Then, at *t*_*f*_ (time since the gear was brought onboard, which ranged between approximately 1 hour and 9 hours), the dead and alive individuals in each container were separated for later identification, counting and length measurement. The time of day, temperature and oxygen concentration of each container were recorded at *t*_*f*_. A random sample of fish remaining alive was video recorded (details provided below).

**Fig 2:**
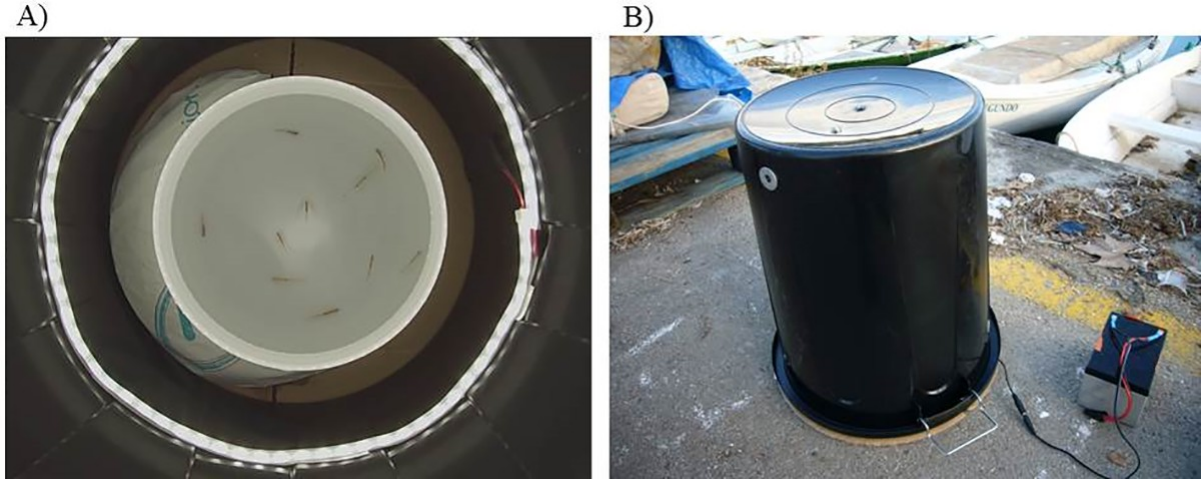
Image of a video recording of ten individuals during the transparent goby fishery sampling **(A)** and image of the structure used for the video recordings **(B)**.

The swimming speed and the reaction to a stimulus of the individuals that came to port alive were estimated using the videos. A sample of ten randomly selected individuals per haul were recorded in a circular arena measuring 23 cm in diameter (Fig 2A). The entire trajectory of the individuals within the arena was recorded using a GoPro HERO 3 camera (GoPro, Colorado, USA). To avoid any lighting interference, the camera was mounted on the top of a larger, upside-down, black structure covering the arena (Fig 2B). Battery powered light-emitting diode lights were also mounted around the camera. The fish were given 5 min to acclimatize to the new container, after which the arena was video recorded for 10 min. The reaction to a stimulus was analyzed by dropping a piece of metal near the arena, which was accomplished using a magnetic release at the mid-time of the 10 min period. After video recording, all the animals were sacrificed and stored for later identification, counting and measurement.

#### Ethics statement

The study was authorized by the Fisheries Department of the Balearic Government. All sampling, experimentation and euthanasia of individuals were carried out in strict accordance with the recommendations of Directive 2010/63/UE, adhering to Spanish law (RD53/2013, BOE n. 34, February 8^th^, 2013). Euthanasia was performed with electrical stunning, and all efforts were made to minimize suffering.

### Data analysis

#### The survival model

**Fig 3:**
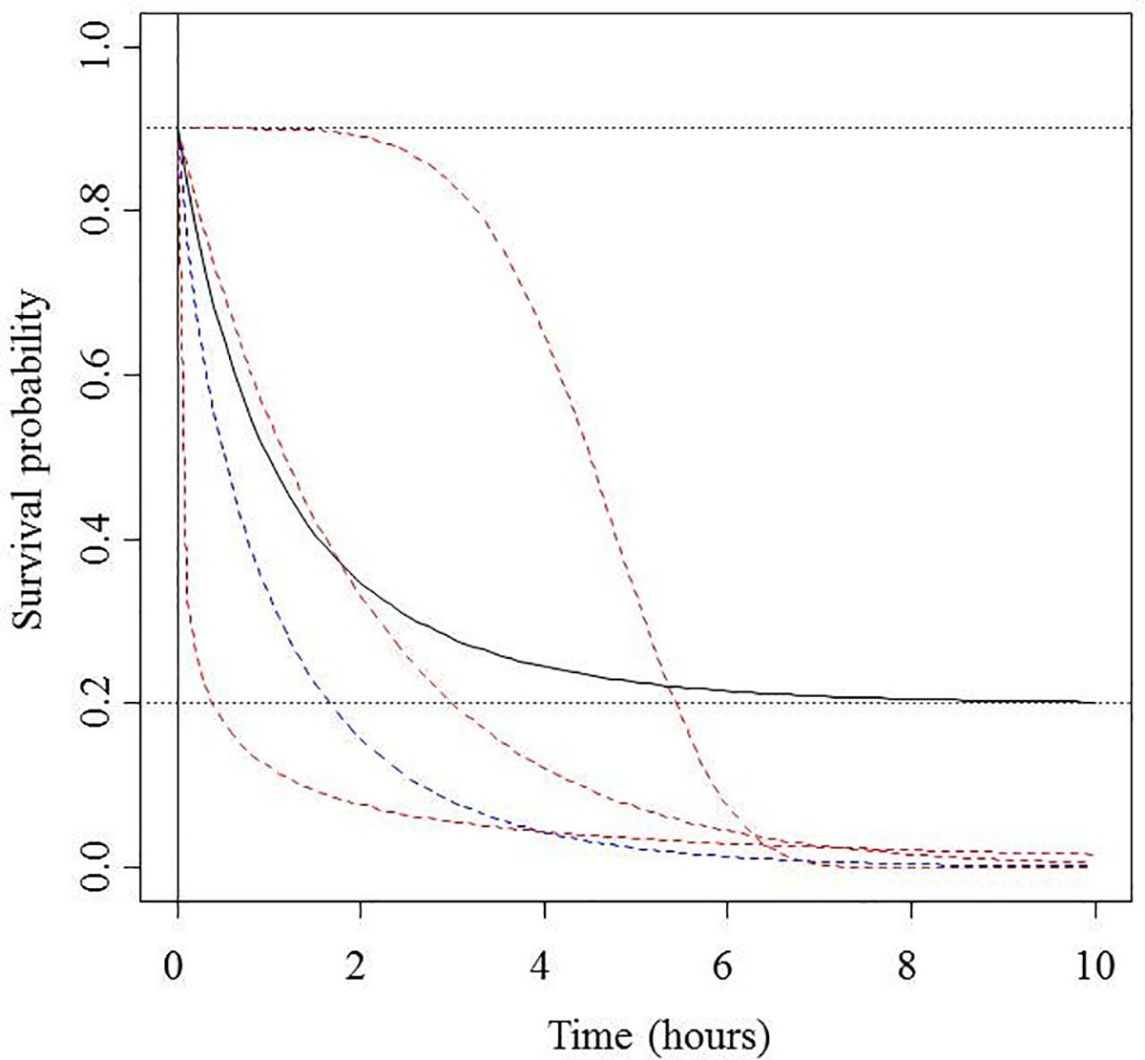
Expected time dependence of the weibull mortality model (with different values of the model parameters; in red) [25], the special case of the negative exponential mortality model (in blue) [25] and the model proposed here (in black).

Preliminary inspection of the variability in the observed survival ratio at different times after the gear was brought onboard (*t* calculated as *t*_*f*_-*t*_*0*_) suggested that the survival probability to time *t* reached an apparently steady state at a level different from zero when *t* was relatively small (Fig 3). Such a pattern can be predicted by neither a conventional Weibull model nor a negative exponential model [25] because in such models, the lower long-term limit is zero survival.

Specifically, the conventional exponential model assumes a constant mortality rate. Conversely, the variation in the number of fish *dN/dt* that are alive after a given time *t* from the time that the gear came onboard may be time varying:

$$\frac{\mathrm{dN}}{\mathrm{dt}}=-m(t)N$$ (1)

where *m(t)* is mortality. The expected number of fish that survive after a given time *t* (i.e., *N(t)*) will be:

$$N(t)=N_{0}e^{-\int_{0}^{t}mt\prime {\mathrm{dt}}^{\prime }}=N_{0}e^{-M(t)}$$ (2)

Certainly, all fish will die at some point if they are kept in captivity for a very long time, but in this case, the estimated mortality will not only be related to fishing practice mortality but also result from the confounding effects of fishing, captivity conditions (e.g., infections or non-natural feeding) and even senescence. According to Gil et al. [26], time variation for the instantaneous mortality rate, *m(t)*, can be modeled as:

$$m(t)=m_{\infty }+(m_{0}-m_{\infty })e^{-t/\tau }$$ (3)

This model seems to be appropriate for describing the biological process of accumulated mortality related to fishing because it is maximal when fish come onboard at time *t* = 0 (*m*_*0*_), *m(t)* decreases toward a smaller asymptotic mortality (*m*_*∞*_) and τ determines the rate of change between *m*_*0*_ and *m*_*∞*_. We propose that in the short term and in the absence of other mortality-related processes, *m*_*∞*_ *=* 0 (i.e., stabilization of *N*), and thus:

$$m(t)=m_{0}e^{-t/\tau }$$ (4)

Solving *M(t*,*t*_*0*_*)* from Eq 2 and Eq 4 yields:

$$M(t)=m_{0}\tau (1-e^{-t/\tau })$$ (5)

Finally, *N*_*f*_, the number of fish remaining alive at any time, will be:

$$N_{f}(t)=S_{0}N_{0}e^{-m_{0}\tau (1-e^{-t/\tau })}$$ (6)

where *S*_*0*_ is the survival probability when the gear was onboard (i.e., the number of live fish from a sample of *N*_*0*_ individuals). Thus, the asymptotic survival (survival at a large *t*) will be given by:

$$N_{f}(t=\infty )=S_{0}N_{0}e^{-m_{0}\tau }$$ (7)

Provided that the survival patterns may be modulated by different drivers, the lineal dependence of *S*_*0*_ and *m*_*0*_ on different covariables was evaluated. Specifically, the covariables considered were:

$$m_{0}=\alpha_{0}+\alpha_{1}\mathrm{Depth}+\alpha_{2}{\mathrm{Temp}}_{f}+{\mathrm{rnd}}_{1}$$ (8)

$$\mathrm{logit}(S_{0})=\beta_{0}+\beta_{1}\mathrm{Depth}+\beta_{2}{\mathrm{Temp}}_{0}+{\mathrm{rnd}}_{2}$$

where *Depth* is the depth at which the haul was operated (which may increase mortality due to barotrauma), *Temp*_*0*_ and *Temp*_*f*_ are the water temperatures at the time the gear was brought onboard and at the time the dead/live fish were counted at port. In addition, a single level of random effects on *m*_*0*_ and on *S*_*0*_ (*rnd*_*1*_ and *rnd*_*2*_, respectively) was considered. Specifically, random (normally distributed with a zero mean and variance *σ*_*1*_ and *σ*_*2*_) variation between the hauls from the same fishing trip was modeled.

The mortality estimated with this model (Eq 5) could be influenced by captivity conditions, given that the use of true control fishes was unfeasible in this study. In such a case, mortality can be decomposed into fishing (*m*_*f*_) and handling (*m*_*h*_) mortality; i.e., assuming additive effects, *M(t)* = *m*_*f*_ + *m*_*h*_. Nevertheless, the relative mortality rates of different groups of fish can be compared even when *m*_*h*_ remains unknown [14]. Here, fishing mortality under a given environmental condition was compared with fishing mortality under a reference environmental condition (*m*_*f*.*reference*_), which was defined at the average depth and temperature (Eq 8). Therefore, the handling mortality cancels out when modeling *e*^*mf+mh*^ */ e*^*mf*.*reference+mh*^ [14]. Accordingly, the slope of any environmental effect on *m*_*f*_ can be properly estimated even when an unknown *m*_*h*_ has an additive effect.

#### Fitting the survival model

The unknown parameters of the survival model were estimated using a Bayesian approach. The number of surviving fish when haul *i* was onboard (*N*_*0*,*i*_) was assumed to be the result of a binomial process of *N*_*i*_ trials (number of dead fish plus number of alive fish when the gear was onboard) with probability *S*_*0*,*i*_ (Eq 8). The number of surviving fish after *t*_*i*_ hours (*N*_*f*,*i*_) was assumed to be the result of a Poisson process with mean *M*_i_*N_*0*,*i*._ This combination of random processes was selected after comparing other combinations of binomial, Poisson and three alternative implementations of negative binomial processes (see Results). The reliability of all these alternative implementations was successfully evaluated via simulations, and the implementation details are provided in the (S1 R script).

A nearly noninformative gamma distribution (shape = 0.01, scale = 0.01) was assumed as a prior for τ and for the tolerance (1/variance) of the two random effects considered (hauls from the same trip; on *S*_*0*_ and on *m*_*0*_). Alternative implementations of these priors (uniform distribution) rendered the same results. Priors for all the parameters of the linear dependencies were assumed to be normally distributed with a zero mean and a very large variance (10^16^). Three Markov chain Monte Carlo (MCMC) chains were run using randomly selected initial values for each parameter within a reasonable interval, and conventional convergence criteria [27] were checked. The number of iterations selected for each run was 60,000 values per chain after convergence and thinning (1 of 50). The model was implemented with the *R2jags* package (http://cran.r-project.org/web/packages/R2jags/R2jags.pdf) in R software (at http://www.r-project.org/), which uses the samplers implemented in JAGS (http://mcmc-jags.sourceforge.net/) to move the MCMC chains. The R script and the input data are supplied as (S2 R script and S1 Input data).

Because the fishery targets two species (*A*. *minuta* and *P*. *ferreri*), preliminary analyses were aimed at estimating species-specific mortality. However, because the models used for those analyses failed to converge (see the Results section) and the two species are managed, fished and marketed together, here, we explore the fishing mortality patterns of the species ensemble.

#### Algorithm for tracking fish trajectories

Manual tracking of object trajectories in a video by observers is a very time-consuming task. Currently, software enhancements for obtaining information about moving objects from videos are being increasingly implemented [28]. In this study, Python (v2.7; https://www.python.org/) code was developed to automatically track fish trajectories, taking advantage of the image analysis functions implemented in the OpenCV library (v3.2; http://opencv.org/). The code is structured as object oriented, and each fish trajectory is treated as an independent object.

The algorithm was applied to each frame of the time studied in each video, i.e., two minutes before and two minutes after the stimulus. The circular arena was the region of interest in the image, and it was detected automatically in each frame using a threshold value in the grayscale image. In the first frame, the initial position of each tracked fish was selected manually. For the remaining frames, the fish was identified as a blob (i.e., a region of the image with a large likelihood of being a fish) in a binary image during the process of image segmentation. Given that illumination was not uniform in the region of interest, an adaptive threshold based on the weighted sum of neighboring pixel values achieved better results for image segmentation than did a unique threshold value. Fish shadows in the arena bottom could produce false fish detections during image segmentation, and only blobs with a certain area were considered. The center of mass for every blob and the Euclidean distance between each blob and each fish position in the previous frame were calculated. To assign a fish to a blob, the Munkres algorithm [29] was applied to the distance matrix between blobs and fish in the previous frame. Overlap between fish or problems in segmentation due to fish transparency or poor lighting conditions can generate a smaller number of blobs than fish. In such cases, the same fish position in the previous frame was assigned to the fish without blob matching, and the fish being tracked was labeled. The position of the fish labeled in such a way was solved in the next frames. The output of the code was the trajectory of all the fish in a video. Fish speed, measured as the difference between two consecutive positions, was extracted for all the frames and all the fish in each video. A few errors in the trajectories were detected when two or more fish overlapped (i.e., occlusions). However, these failures did not have a relevant impact on the mobility metrics because the average mobility among all the fish in any given video was estimated. Although other codes for fish tracking have been developed [30], the lighting conditions and the transparency of the fishes forced us to implement specific software for this study.

#### Analyzing vitality

The position of each fish in every video frame was estimated from two minutes before until two minutes after the stimulus. The speed in each frame (*v*, mm/second) was extracted from those trajectories. To minimize temporal autocorrelation, the size of such speed data was reduced by averaging over 60 consecutive frames. Given that the frame rate was 20 frames per second, the time series actually analyzed for a given fish was composed of 40 speed measures before and 40 measures after the stimulus. The speed of fish *i* at moment *t* (*v*_*i*,*t*_) was assumed to be gamma distributed as:

$$v_{i,t}∼\mathrm{Gamma}({\mathrm{shape}}_{i}{,\mathrm{rate}}_{i})$$ (9)

$$\mathrm{where}\;{shape}_{i}={\bar{v}}_{i,BA}^{2}{tol}_{i,BA}\;\mathrm{and}\;{rate}_{i}={\bar{v}}_{i,BA}{tol}_{i,BA}$$ (10)

where ${\bar{v}}_{i,BA}$ and *tol*_*i*,*BA*_ are the fish-specific (*i*) and period-specific (either before or after the stimulus) mean speed and tolerance (i.e., inverse of the variance), respectively, and

$${\bar{v}}_{i,BEFORE}=v_{mean.BEFORE}+{\Delta B}_{i}$$ (11)

$${\bar{v}}_{i,AFTER}=v_{mean.BEFORE}+\Delta BA+{\Delta A}_{i}$$

where ${\bar{v}}_{i,BEFORE}$ is the average speed of fish *i* before the stimulus, *v*_*mean*.*BEFORE*_ is the average speed of all the fish from a given haul before the stimulus, *ΔB*_*i*_ is a fish-specific random effect before the stimulus, ${\bar{v}}_{i,AFTER}$ is the average speed of fish *i* after the stimulus, *ΔBA* is the speed difference between *v*_*mean*.*BEFORE*_ and the average speed of all the fish from a given haul after the stimulus, and *ΔA*_*i*_ is a fish-specific random effect after the stimulus.

The two random effect levels considered are defined as:

$${\Delta B}_{i}∼N(0,{tol}_{B})$$ (12)

$${\Delta A}_{i}∼N(0,{tol}_{A})$$

The parameters of this model were fitted using a Bayesian approach with noninformative priors and the same technical settings described above. *tol*_*i*,*BA*_, *tol*_*B*_ and *tol*_*A*_ were assumed to be gamma distributed, and *v*_*mean*.*BEFORE*_ and *ΔBA* were assumed to be normally distributed with a zero mean and a very large variance. An R script with the code for completing the analyses and the data input for one fish are provided as (S3 R script and S2 Input data).

#### Relating survival to vitality

The relationships between the estimated asymptotic post-release survival rate and the vitality metrics were assessed using a simple linear model where the response variable was the logit-transformed survival rate (which was estimated as described above) and the putative explanatory variables were the two vitality metrics (*v*_*mean*.*BEFORE*_ and *ΔBA*), *t* (hours since the gear was brought onboard), *Depth* and *Temp*_*f*_ (i.e., the water temperature at port arrival). The best model in terms of the Akaike information criterion was selected using the *step* function in the R library *Stats*.

## Results

### Species specificities

**Fig 4:**
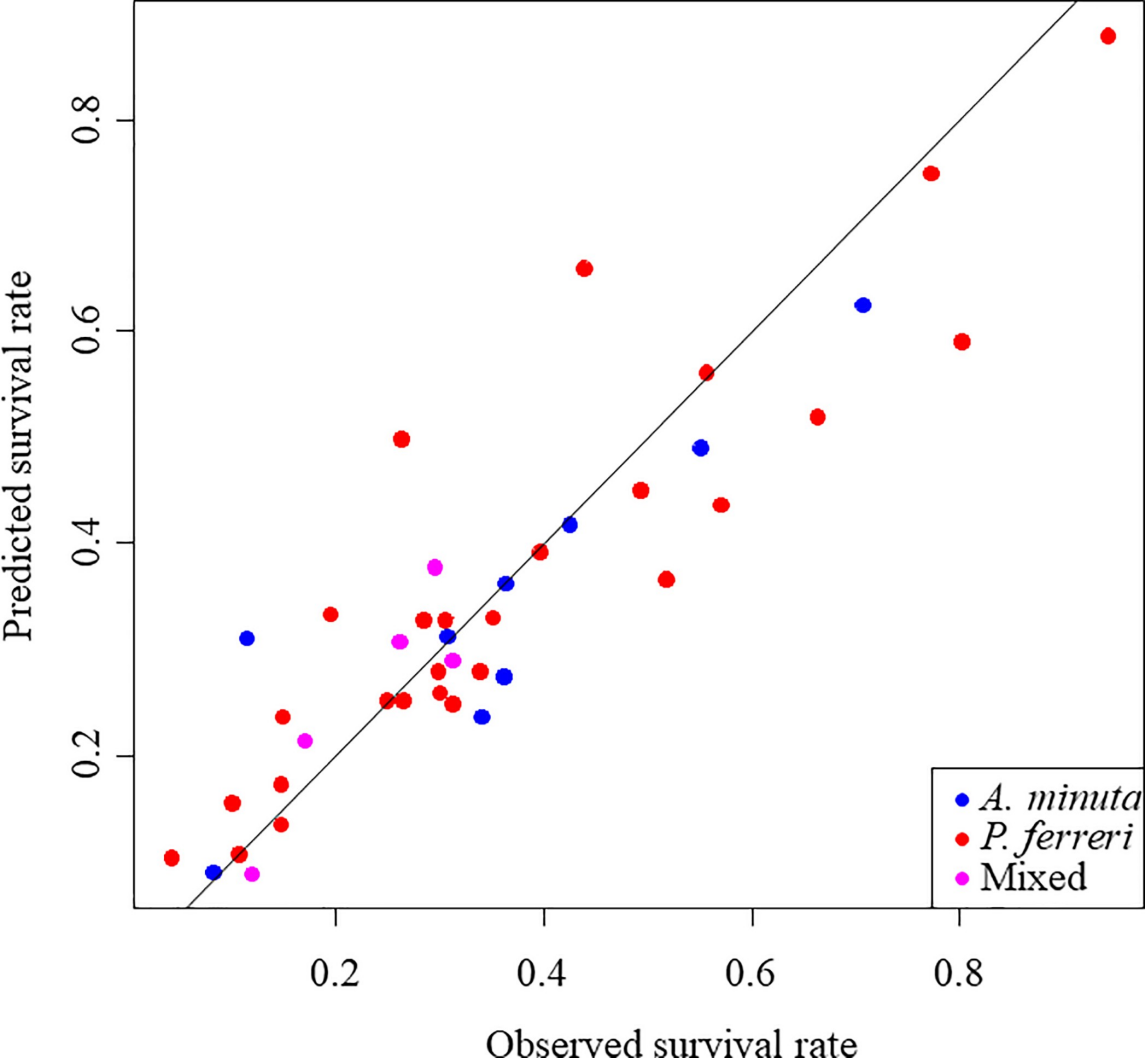
Observed versus expected survival rates. The line has a slope = 1 and an intercept = 0. Hauls have been classified into three categories according to the prevalence of the two species considered.

As mentioned above, a preliminary attempt to fit a species-specific survival model was made. The catch of a given haul was classified into one of three categories according to the fisher's expertise: 1) more than 90% of *A*. *minuta*, 2) more than 90% of *P*. *ferreri* or 3) mixed catch. However, the small number of species-specific hauls precluded any attempt to estimate species-specific mortality (the models failed to converge). Nevertheless, the environmental variables considered did not differ between categories 1 and 2 (one-way analysis of variance (ANOVA) for depth: *P* = 0.877; ANOVA for temperature when the haul was onboard: *P* = 0.825; and ANOVA for temperature at port: *P* = 0.121). Therefore, species composition seems unbiased in relation to the environmental variables considered. Moreover, the observed and expected patterns of mortality (Fig 4) did not suggest the existence of any species-specific bias or trend. Therefore, thereafter, hauls were pooled and species composition was ignored because the implicit assumption that fishing mortality patterns would be similar for both species seemed plausible. Thus, given that the two species are managed, fished and marketed together, here, we explore the fishing mortality patterns of the species ensemble.

### Survival

**Fig 5:**
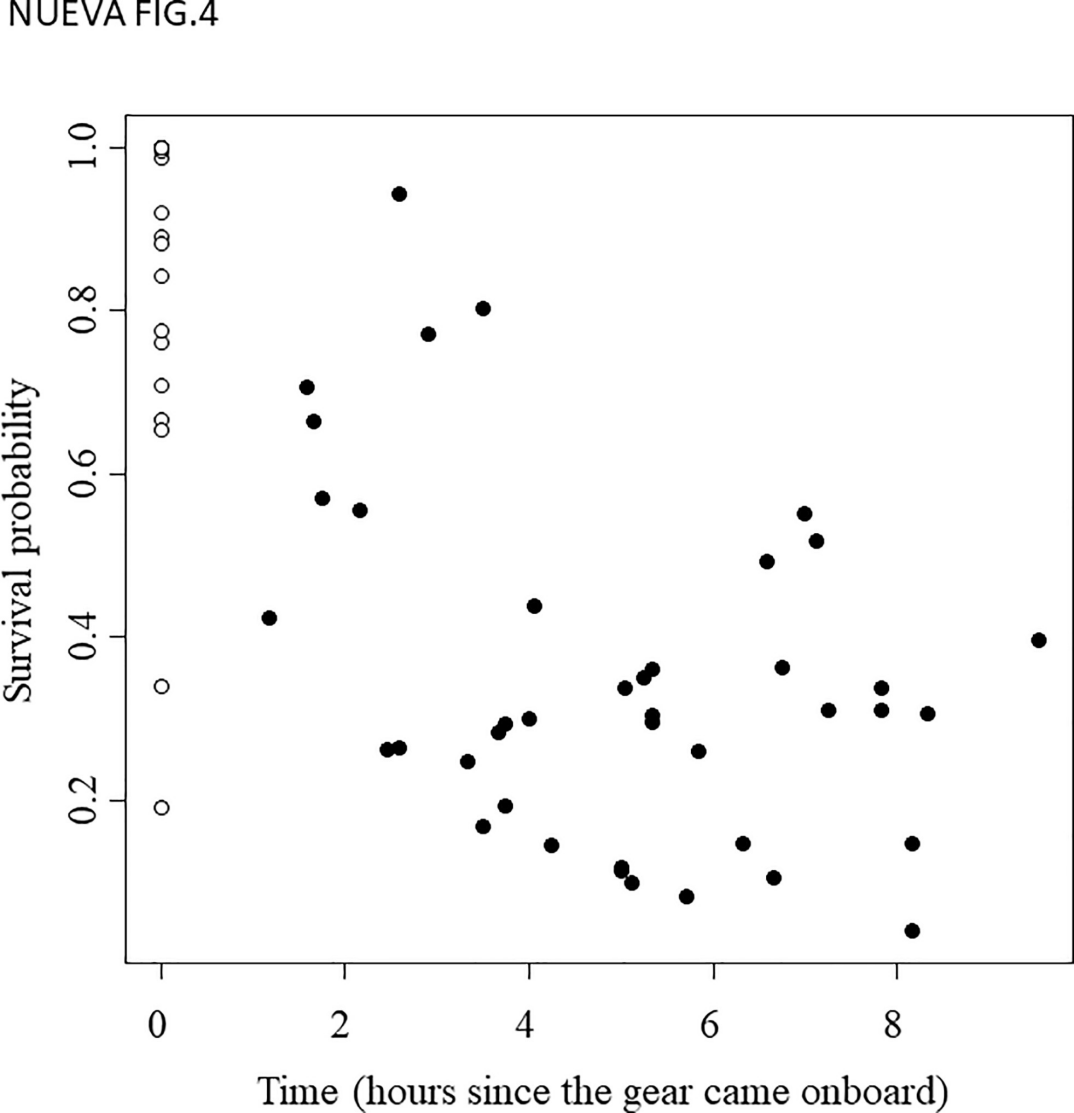
Raw survival probability (i.e., before accounting for the effects of covariables). Each white point represents a haul at *t*_*0*_ (i.e., the time when the gear was brought onboard), and each black point represents a haul at *t* (i.e., the time since the gear was brought onboard). The survival probability is estimated as the ratio between the number of fish that were alive and the total number of fish surveyed.

Six of the 47 sampled hauls were excluded from the survival analyses because the oxygen concentration of the water container registered at port arrival was too low to guarantee that the handling mortality and the fish mobility were comparable to those of the other sampled hauls. The average number of fish sampled per haul was 189.6 ± 84.5 (mean ± sd). The average number of fish that were alive when the gear came onboard was 176.2 ± 89.0, which corresponded to an average percentage of 91.7%. The average number of fish remaining alive when they arrived at port (i.e., after *t* hours since the gear was brought onboard, being *t* = 4.9 ± 2.1 hours) decreased to 64.4 ± 47.9. The average percentage of fish remaining alive when *t* ≥ 4 hours after the gear came onboard was only 27.8%. Therefore, the observed survival rate appeared to be close to 100% just at the time when the gear was brought onboard, after which it quickly decreased and seemed to converge to a steady value after a few hours (Fig 5); thus, the observations seem to fit the conceptual model in Fig 3.

The pooled data were successfully fitted to the survival model (Eqs 6 to 8). The binomial (for *N*_*0*_) and Poisson (for *N*_*f*_) processes rendered the best results in terms of deviation information criteria. Noticeably, all of the implementations with negative binomial distributions (S1 R script) failed to converge. The median and 95% Bayesian credibility interval of parameters from the model are detailed in Table 1. Note that temperature seems to negatively affect only delayed survival (*m*_*0*_), and depth seems to negatively affect only immediate survival (*S*_*0*_).

**Table 1:** Estimates of the survival model parameters.

Parameter	2.5%	Median	97.5%
*m*_*0*_*α*_*0*_ (Intercept)	0.177	0.298	0.429
*m*_*0*_*α*_*1*_ (*Depth*)	-0.002	0.0084	0.019
*m*_*0*_*α*_*2*_ (*Temp*_*f*_)	0.059	0.094	0.142
Logit (*S*_*0*_) *β*_*0*_ (Intercept)	3.410	5.520	8.850
Logit (*S*_*0*_) *β*_*1*_ (*Depth*)	0.025	0.066	0.109
Logit (*S*_*0*_) *β*_*2*_ (*Temp*_*0*_)	-3.560	-0.824	1.780
*Τ*	1.930	2.570	3.470
*σ*_*1*_ (random effect on *m*_*0*_)	0.123	0.182	0.286
*σ*_*2*_ (random effect on *S*_*0*_)	2.508	4.072	7.809

**Fig 6:**
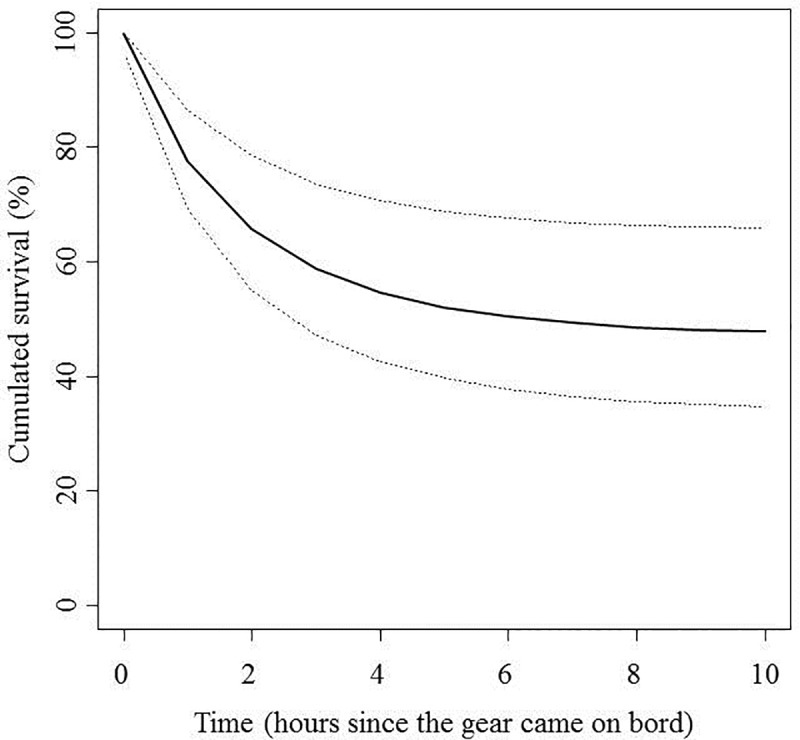
Expected survival probability (in %) for fish from a haul deployed under the average environmental conditions (depth and temperature). Thi**s** is the reference pattern chosen for estimating relative survival.

This model reasonably explained the observed values (i.e., counts of live fish /total fish after *t*_*i*_ hours; see Fig 4). In the case where no mortality source other than fishing mortality was in effect, the expected immediate survival probability for fish captured at the average sampled depth (25.7 m) and temperature (15.7 ºC) was 99.9% (95% credibility interval (CI) between 97.9% and 100%; Fig 6 and Eq 7), but the asymptotic delayed survival when fish were immediately released when the haul was onboard was 47.2% (95% CI between 33.8% and 65.8%). Note that this expected delayed survival is higher than the average observed survival rate (27.8%) because to emulate immediate release, *Temp*_*f*_ (the temperature of the container at port arrival) was set to equal *Temp*_*0*_ (water temperature of the sea), which is always lower, and increasing temperature negatively affects delayed mortality (Table 1).

**Fig 7:**
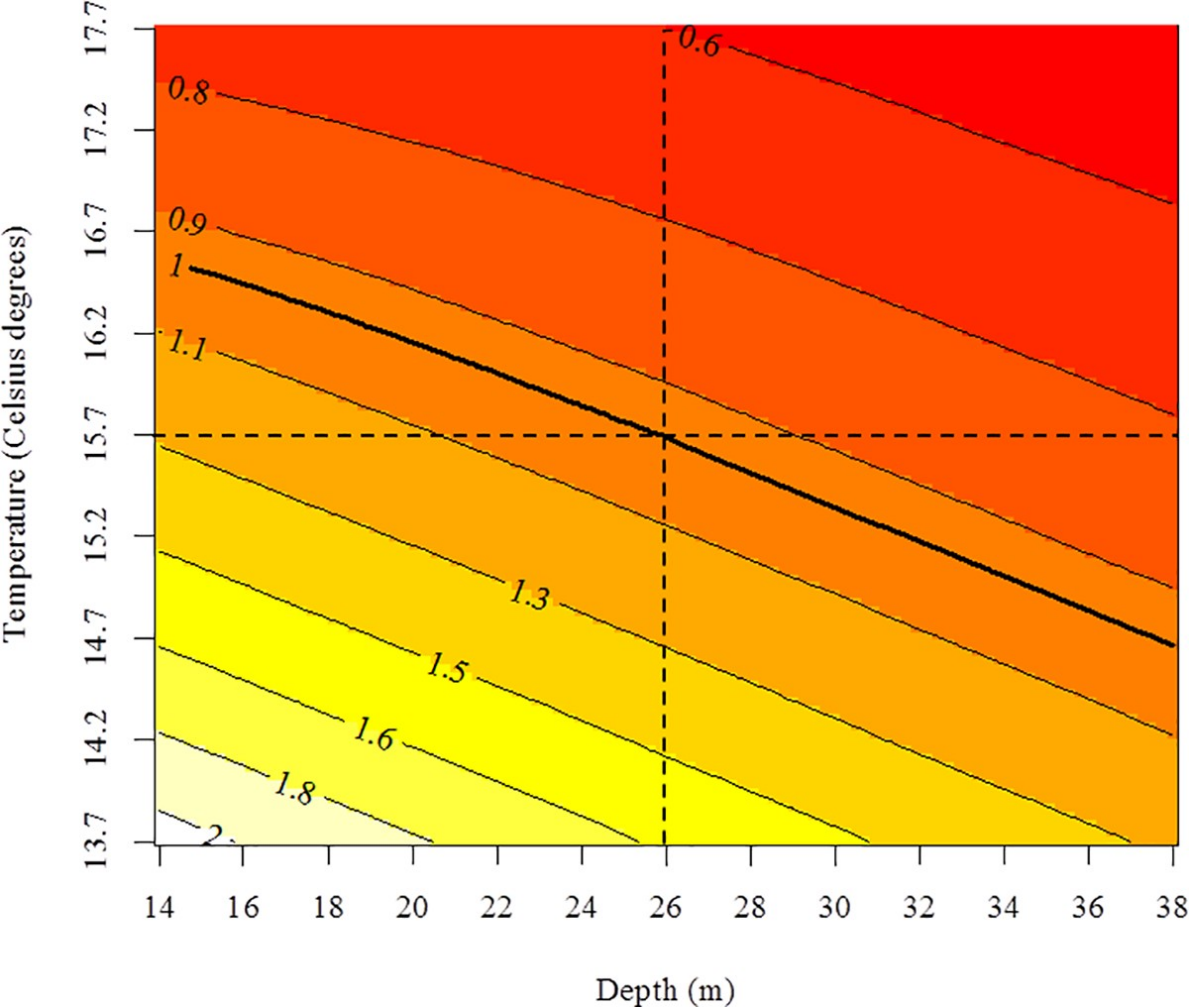
Estimated relative survival rate under different combinations of environmental variables (depth and temperature). The reference level was a haul deployed under the average environmental conditions (represented as the dashed lines). The thick isoline represents the environmental combinations for which the expected fishing survival was the same as that for the reference level. The thin isolines represent the environmental combinations for which the expected fishing survival was the same regarding the reference level. For example, the isoline of 1.5 represents the environmental combinations for which it is expected that the survival rate will be 50% higher than that for the reference.

As mentioned above, these figures should be interpreted with caution because fishing mortality and handling mortality could be confounded. Nevertheless, the fishing mortality pattern relative to a reference (here, a haul deployed under the average environmental conditions of depth and temperature) provides unbiased estimates of fishing mortality due to environmental drivers. Thus, to facilitate the interpretation of the model predictions, the expected values of relative survival were estimated under different combinations of environmental conditions (depth and temperature) within the sampled ranges (Fig 7).

The resulting pattern suggests a relevant effect of both depth and temperature in such a way that a similar survival probability may result from either a warmer temperature in shallower waters (e.g., 16.2 ºC and 16 m) or a colder temperature in deeper waters (e.g., 14.5 ºC and 35 m). At the average depth, a temperature change of 2.8 ºC should either halve or double the expected fishing survival. At the average temperature, a depth up to 32 m should double the expected fishing survival. Changes of 10 m in depth would cause changes of approximately 20% in expected fishing survival.

### Vitality

**Fig 8:**
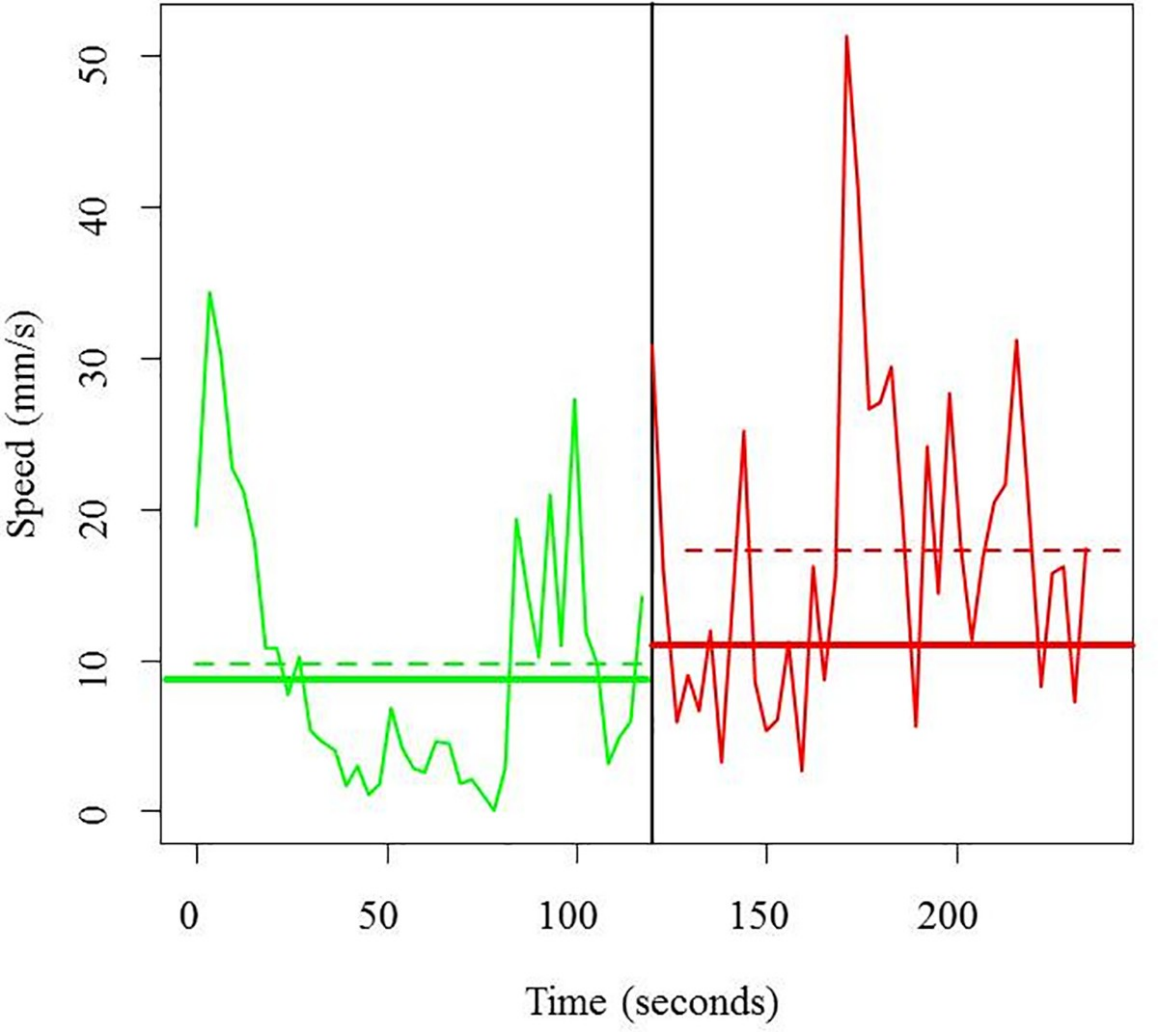
Speed (in mm/s) of a given fish during 4 min of video recording. The black solid vertical line indicates the moment at which the sonorous stimulus occurred. The green solid horizontal line indicates the mean speed of all fish in a haul before the stimulus (*v*_*mean*.*BEFORE*_), the green dashed line indicates the mean speed of a given fish in a haul before the stimulus (i.e., ${\bar{v}}_{i,BEFORE}$), the red solid line indicates the average speed of all the fish from a given haul after the stimulus, and the red dashed line presents the mean speed of a given fish after the stimulus (i.e., ${\bar{v}}_{i,AFTER}$). The distance between the green solid line and the green dashed line represents the parameter *ΔBi*, the distance between the red solid line and the red dashed line represents the parameter *ΔAi*, and the distance between the green solid line and the red solid line represents the parameter *ΔBA*.

The object-tracking algorithm successfully estimated the individual trajectories of the fish (S1 Video). As an example, the speed deviations of a given fish over 4 min are shown in Fig 8, where the parameters of the analyzed model are visually depicted. The Bayesian model (Eqs 9 to 11) successfully extracted the two vitality metrics (*v*_*mean*.*BEFORE*_, mean speed of all fish in a trial before the stimulus, and *ΔBA*, speed increase after the stimulus; Fig 8).

**Fig 9:**
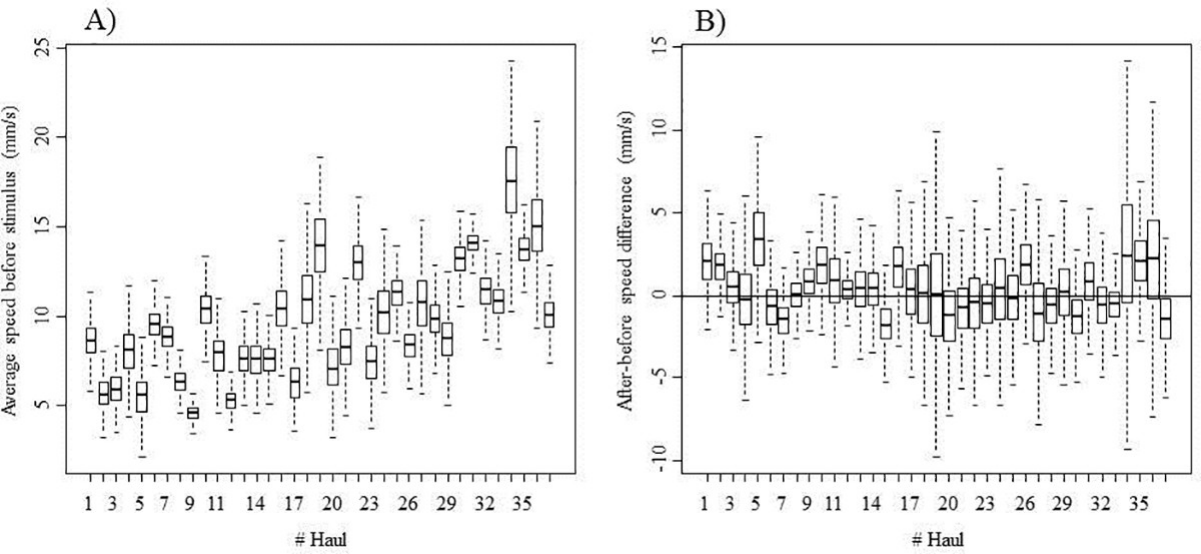
Boxplot of the mean speed of all fish before the stimulus, *v*_*mean*.*BEFORE*_ **(A)**, and boxplot of the before-after speed difference, *ΔBA* **(B)**, for each of the 37 recorded videos. A line within the box marks the median values, the boxes denote the 25 and 75% percentiles, and the bars represent the minimum and maximum nonoutlier values, respectively.

The (between-haul) average of the (between-fish of a given haul) average speed was 9.5 mm/s, but the between-haul variability was large (95% quantile: 5.2 to 15.0 mm/s; Fig 9A). In some hauls, fish increased their speed after the stimulus, but in most of cases, the 95% CI for *ΔBA* included zero; thus, the before-after difference was not significant (Fig 9B).

### Survival vs vitality

Finally, after regressing the survival rate on several putative explanatory variables, the best model (Table 2) was composed of one of the vitality metrics (*v*_*mean*.*BEFORE*_, mean speed of all fish in a haul before the stimulus) and the depth and water temperature when the gear was brought onboard and when motility was evaluated.

**Table 2:** Coefficients of the explanatory variables in the survival model.

	Estimate	Std. Error	*t* value	*P* value
Intercept	-1.571	0.319	-4.916	2.95e-05 ***
*v*_*mean*.*BEFORE*_	0.098	0.030	3.307	0.002456 **
*Depth*	-0.071	0.016	-4.398	1.27e-04 ***
*Temp*_*f*_	-0.190	0.058	-3.259	0.002784 **
*Temp*_*0*_	-0.335	0.155	-2.162	0.038702 *
*T*				N.S.
*ΔBA*				N.S.

## Discussion

### Mortality of the target species

The present study introduces an approach for assessing the relative post-release mortality of two target species (*A*. *minuta* and *P*. *ferreri*) in the transparent goby fishery and the environmental drivers of post-release fish mortality. Usually, these species are returned to the sea when the maximum daily catch for a boat is exceeded or when the fraction of the catch to be discarded is large, which technically hinders fish sorting and promotes slipping. In these cases, the survival of released fish remains unknown, underestimating the total mortality caused by fishing. This underestimation may affect the proper management of the fishery, which is based on the periodic review of daily catches and fishing effort [24]. The immediate mortality estimated in this study under average environmental conditions was low (average survival percentage of 99.9%, CI: 97.9–100%), but the asymptotic delayed mortality was considerably high (average survival percentage of 47.2%) and highly variable (CI between 33.8% and 65.8%).

However, any post-release fishing survival estimate involving some form of captive observation can be confounded by captivity-related mortality [5]. Therefore, captivity-related mortality must be disentangled from genuine fishing-related mortality, which may be achieved by, for example, simultaneously monitoring captive fished and control individuals obtained by methods that ensure the lack of any deleterious fishing-related effects [13]. However, it is very difficult to find a true control capture method resulting in no mortality [14]. Due to the small size, fragility and behavioral ecology of the targeted species in the transparent goby fishery, it is virtually impossible to obtain a sample free of any of the effects of fishing. In the cases for which it is not possible to obtain true control fish, a generalized model can be used to compare the relative survival rates of different groups of fish, for example, fish caught using different capture methods or at different depths [14]. In this way, true replicates of fish are used to obtain a correct measure of variation for survival rate estimation. In this study, the replicates (i.e., the hauls) were obtained over a range of capture depths and water temperatures, which guaranteed an adequate design and, consequently, precise evaluation of the putative drivers of post-release mortality.

The survival of released fish may be affected by several biological processes, such as barotrauma, temperature shock, desiccation/air exposure/anoxia, light-related damage to the eyes, trauma related to handling or alteration of antipredator behavior [6, 9, 19]. In the case of the target species in the transparent goby fishery, desiccation/air exposure/anoxia seems to be irrelevant because fish from the codend are immediately released into a barrel with sea water for sorting or are collected using a hand net with the net still in the water (the target species tend to remain near the surface, which facilitates sorting) [24]. Furthermore, in the case of slipping, fish are directly released to the sea; thus, mortality related to desiccation/air exposure/anoxia or even to onboard handling should be considered negligible. Conversely, water temperature seems to have a major effect on survival. A change of 2.8 ºC in average temperature is expected to halve or double survival. The use of any fishing gear causes some degree of stress to fish, which is increased by interactions with several stressors, such as water temperature [6]. The importance of temperature for the survival of discarded species has been shown in several studies at temperate and tropical latitudes [31–33]. Temperature controls virtually all physiological functions and plays a major role in the life history of all species by exerting a critical influence on growth, metabolism, reproduction, distribution, behavior and, ultimately, survival [34, 35]. Additionally, the levels of dissolved oxygen are depressed at higher water temperatures, which may cause additional physiological problems [36].

Depth may play a role complementary to that of temperature in the survival of target species of the transparent goby fishery. However, the effect of depth seems to be smaller because a change of 10 m in depth would cause changes of approximately 20% in expected fishing survival. The effect of changes in depth, which are accompanied by differences in pressure, temperature, and light conditions, on mortality increases with capture depth [6, 37]. In fact, this variable has been identified as the best predictor of release mortality in the majority of deepwater studies because of the injuries produced by changes in pressure (barotrauma) [37]. However, in this study, the effect of depth was smaller than the effect of temperature, likely because the studied transparent goby fishery is located in relatively shallow waters (less than 30 m depth). In fact, current regulations do not allow fishing at depths greater than 30 m (Decree 19/2019, BOIB n. 35, March 16^th^, 2019). Therefore, the survival of the target species will likely be higher than half of the reference value at all the water temperatures occurring during the fishing season (see Fig 7).

Another factor that could affect the survival of the target species is their small body size. Size-specific mortality of discards, with smaller fish showing greater mortality, has been demonstrated in many studies [38, 39]. The increased sensitivity of smaller fish is generally attributed to fatigue from swimming and greater injury from abrasion [6]. Additionally, core body temperature warms faster in smaller fish, making them more sensitive to temperature increases [32, 40]. Therefore, *A*. *minuta* and *P*. *ferreri*, given their small body size, with an average value of 2.34 ± 0.5 cm, could be considered fragile species with high sensitivity to fishing effects.

### Technical innovations in survival estimation

Vitality metrics (i.e., those related to swimming speed or reaction to a sound stimulus) may provide a new method for estimating post-release mortality, thus precluding the need for long-term experiments. A very simple device can be implemented for video monitoring of fish trajectories. Several motion metrics, such as fish speed at any moment and fish responses to a stimulus, can be automatically extracted in real time from the videos. Here, we empirically demonstrated that a motion metric is correlated with post-release survival. Therefore, vitality metrics, once calibrated with survival likelihood estimates, can be used as mortality predictors. In this study, the swimming speed of the target species was correlated with post-release survival and thus could be considered a genuine vitality metric. However, reaction to the sound stimulus did not present a clear relationship with survival. The relationship between some reflexes and post-release mortality has been proven to be species-specific; i.e., all fish have reflexes, but these reflexes differ among species [15]. For example, the vestibular-ocular response was identified as an impaired reflex in sockeye and pink salmon but not in coho salmon [41]. Reflexes are neurological responses to external stimuli, although the synergistic effects of different stressors on reflex impairment are not yet fully understood. Therefore, future research that uncovers the links between physiological disturbance, reflex impairment and post-release mortality would be valuable for different species from both an applied and a fundamental perspective.

### Implications for management

Ignoring the post-release mortality of discarded target species could be a relevant concern for the transparent goby fishery since this fishery is managed based on the catches per unit of effort from the previous month [24]. Unfortunately, due to the impossibility of obtaining genuine control fish in this fishery, an estimate of absolute fishing-related post-release mortality remains elusive. However, the survival reported here could be considered a proxy for the lower bound of survival probability, as long as the captivity conditions have a negative effect (but see the discussion of the predation effects below). In such a case, the environmental drivers reported here still have management implications. For example, survival is expected to be higher than the reference survival at any depth when the temperature is approximately 15 ºC or lower. This temperature corresponds to those observed during most of the fishing season (February to March). Conversely, during the rest of the fishing season, the water temperature is > 15 ºC, and thus, the survival rate will be lower than the reference value, especially when fishing takes place at deeper locations. In such cases, the survival rate could be < 50%, depending on the effect of unknown handling mortality.

Therefore, as a rule of thumb, during the colder months (February to March), high survival is expected for the discarded target fishes, and return to the sea would be advisable because many of the released fish would contribute to enhancing population dynamics. However, during the warmer months of the fishing season, survival is expected to be low. In this case, other measures are recommended. Some fishers, when the maximum daily catch is exceeded, share the catch with other boats already fishing that have not met the maximum [24]. The fishers claim to carry out this practice when the fish are mostly dead (probably during the warmer months), optimizing effort and maximizing the use of the resource. The results reported here support this practice because the target species show high mortality under certain environmental conditions. Therefore, recently, due to the fishers’ request and based on the results of this study, the local administration modified fishing regulations, allowing the practice of sharing the catch (Decree 19/2019). Accordingly, each pair of boats that wish to associate in order to share their excess catches must communicate this wish before the start of the fishing season. This practice is in accordance with the EU discard policy (Regulation EU No 1380/2013 of the European Parliament and the Council on the Common Fisheries Policy), which aims to reduce the number of discards returned to the sea. In fact, the practice of sharing allows for a reduction in the discards of a fishery that, due to the high sensitivity of the target species, suffers high mortality from the fishing process and, in turn, increases the profitability of fishing.

In any case, it should be noted that the rules of thumb outlined above do not take into account that captivity conditions could fail to account for other factors, for example, predation mortality. Predation could be promoted by deficits in orientation, swimming ability, feeding, schooling, social interactions, and predator evasion, increasing discard mortality [40, 42]. Therefore, further investigations should be carried out to obtain more reliable post-release mortality estimates.

In conclusion, the survival model proposed in this study reasonably explains the observed post-release mortality of the transparent goby fishery. Moreover, once the depth of capture and the water temperature are known, it is possible to estimate the delayed relative mortality of the target species by video recording the fish caught for a few minutes. This methodology can be quickly applied onboard and at a relatively low cost, although it is limited to relatively small species. Here, we have demonstrated the utility of this approach for a specific fishery, but it may be applicable to a wider range of fisheries for which discarding is a significant issue.

## Supporting information

S1 Video Fragment of a video recording where the estimated fish trajectories are shown. (AVI)

S1 R script Simulations of the survival model. (R)

S2 R script Survival model. (R)

S3 R script Vitality model. (R)

S1 Input data Survival model. (RDATA)

S2 Input data Vitality model. (RDATA)
